# The School Doctor's Outfit

**Published:** 1915-08-07

**Authors:** 


### THE SCHOOL DOCTOR'S OUTFIT.*

### II.—The Contents of the Doctor's Bag.

WOODEN SPATULAS.—I generally carry half-a-dozen of these, although they are always supplied at the schools, and those in the bag are hardly ever called into use. A celluloid or glass spatula is less useful, for it requires sterilisation, whereas these wooden slips are burned after use.

Pocket Electric Lamp.—This is a most useful instrument, and with one charge lasts about three months, being frequently used during that time. Since I have been in the habit of using a Hay-Flatau pharyngoscope I have discarded the electric lamp.

Urine Testing Apparatus.—This consists of a small leather case containing a diminutive securely capped spirit lamp. The cap is screwed on, and, as a matter of fact, the lamp has been improvised from an ordinary metal capped medicine bottle, such as nurses carry in their bags; and a dozen endolytic urine test tubes. I found these tubes most useful in emergency urine testing, such as is done at schools; they are portable, accurate, and give no trouble whatever. All that is required is to draw up some of the urine into the tube and to heat the tube over the flame. They are supplied in several varieties for testing the presence of sugar, albumen, and decomposition products.

A couple of Swab Tubes, with platinum loop on glass handle, and envelopes for posting them to headquarters for examination and report.

A Sheaf of Perchloride Papers and a small Bottle of Tannic Acid Solution for taking impressions of flat feet.—A swab to apply the tannic-acid solution to the soles of the feet is easily improvised from a pledget of lint or cotton wadding supplied by the nurse, who generally carries some first-aid equipment in her bag. By means of these papers permanent records of cases of flat feet can be obtained and preserved with the record card of the child examined.

A Dynamometer, children's size, with spring adjusted to 50 kilos. maximum.—This is for special investigations and can hardly be considered a necessity.

So, too, is the Martin's Riva Recci Sphygmomanometer, with children's armlet, and non-spillable tube.—This is used for blood-pressure investigations and fits nicely into the bottom of the bag.

Ophthalmoscope.—I use a Morton's instrument, but the opportunities for adequately using it are few and far between.

Test Types.—These are supplied at every school, and are, therefore, not necessities to be carried in the bag. For emergencies, however, I carry a folding Snellen's test-card, an astigmatic dial-card, and a small Jaeger vision test-card for testing near vision in doubtful cases of hypermetropia. All these have been found exceedingly useful.

Hay-Flatau Pharyngoscope.—This very useful instrument I have only lately added to my armamentarium, but I regard it as a distinct boon. It enables the inspector to observe the pharynx and naso-pharynx with great convenience and comfort to the child; it is a handy and simple diagnostic instrument which should be more widely known than it appears to be. Light is supplied by a small pocket battery, and the whole apparatus is easily carried in one of the side pockets of the bag.

Laryngoscope.—I carry a set of mirrors, a frontal mirror with fixation band, a thin nasal forceps, several Thudicum's specula, and a nested set of aural specula, and these I find quite sufficient for all routine inspections. Since using the Hay-Flatau apparatus the laryngoscopic set has very rarely been needed. A thorough inspection of the interior of a child's ear can hardly be carried out at the school, but must be left for a later occasion when the child is seen at the treatment centre.

### MY NOTEBOOKS.

The side pockets of my bag contain a small supply of advice cards and two notebooks, one for boys and the other for girls. It is a good rule to keep a record, for private use, of all cases seen. Normal children need not be entered upon the book, but all abnormal or defective children should be noted. My notebooks, kept for several years back, have been most useful, and have given me data for several papers on special subjects which would otherwise have had to be laboriously collected from the record cards—a task that needed frequent private visits to the schools concerned.

The habit of making notes cannot be too strongly recommended to all medical inspectors. It may be argued that enough time and energy is consumed in writing up the records, but to make a brief note of a defect discovered at the routine examination demands very little additional time and energy, and that expenditure is amply compensated afterwards when data are required for some special investigation.

In addition, I carry in my bag a small bottle of iodine solution, which is frequently renewed. It is a very useful antiseptic, and comes in handy on numerous occasions. Lately, however, I have discarded the iodine in favour of a 2-per-cent. solution of picric acid with some thymol added to it. This solution keeps better, is as efficient an antiseptic as iodine solution, and does not stain to the extent the latter does. A nail-brush and an aluminium soap-box, with a cake of superfatted soap, complete the outfit.

### WEIGHT AND CARE OF THE INSTRUMENTS.

To this armamentarium one may add according to one's taste. The bag is big enough to contain far more material, but the instruments mentioned above are adequate for all ordinary routine work, supplemented as they are by what the school nurse carries in her bag. I would add a lens measurer to test the glasses worn by children who have been recommended for glasses, and perhaps a small

* The first article appeared in THE HOSPITAL of July 31, p. 378.

outfit for taking smears for microscopical examination later on. Where open-air schools are inspected a small outfit for hæmoglobin estimation may also be carried, but this is more in the nature of a special investigation, and can scarcely be regarded as ordinary equipment.

Fully loaded my bag weighs rather heavy, but it is a compact and serviceable *vade mecum* that one cannot afford to dispense with, and it is not often that one requires to carry it any distance. Those who do not possess the advantage of a motor-car have always the use of a tramcar or 'bus to reach their destination, and the little extra weight of the bag does not matter much.

A word must be said about the care and preservation of the instruments. Such an outfit as I have endeavoured to describe is comparatively expensive, but it will last a lifetime if it is properly looked after. The instrument most frequently in want of repair and renewal is the binaural stethoscope: the indiarubber tubes do not last very long, and the steel framework gets less and less attractive-looking every month, so that renewal is frequently required. I have not found any enduring satisfaction in using steel flexible tubes, but ribbed rubber seems to last longer than the ordinary smooth kind and gets less foul. All the metal instruments require to be rubbed up once every month and carefully overhauled. There is no great advantage in having them silver-plated; it merely adds to the expense without adequately enhancing their utility. L.R.C.P.

